# On the Use of the Turn-Key

**Published:** 1839-09

**Authors:** S. M. Shepherd

**Affiliations:** Petersburg, Virginia.


### ON THE USE OF THE TURN-KEY.

[ BY S. M. SHEPHERD, PETERSBURG, VIRGINIA. ]

It is a matter of regret, that there has been, and still is, so much stiffness and selfishness among our profession. It seems that they are almost afraid to talk to each other on the subject of the Dental Art, each one fearing he will impart to the other some information he never *had* before. Or, it may be, that each one fears he will expose his ignorance. In either case the matter is wrong, and ought to be corrected. As all now have an opportunity through this Journal of giving their views, on different points, it is to be hoped that the liberal-minded will not be backward. By way of commencement, I propose to give a few thoughts on the use of the turn-key. It seems from various remarks in the late books and papers on the subject of the extraction of the teeth, that most of the profession have laid aside the key almost entirely. I believe a great deal depends on the skill of the operator in the use of any instrument to make it efficient. He may use forceps until he can overcome most of the common difficulties without the aid of any other instrument. But all unprejudiced men will confess that the objects are to remove a tooth with facility, and at the same time to avoid unnecessary pain. Then I contend that he who has the ability to accomplish this object with the key, ought not to exchange it for any other instrument. For if he chooses any other, he cannot do more than obtain the above named object: and he necessarily inflicts much useless pain in making the change. That the key possesses great power, no one can doubt who has been in the habit of using it: and that many have had the mortification of seeing miserable wounds inflicted on the gums and jaws of their patients is equally true. This no doubt, has caused many feeling men to lay aside that powerful instrument, thinking the injuries were a necessary consequence of the use of it. Any man of proper feeling would, under similar circumstances, come to the same conclusion. But I believe that no avoidable injury is produced by a proper use of the key. If the instrument is improperly proportioned, no man can use it with good effect; but if the proportions are correct, and the operator skilful, I believe it is the most certain, safe, and least liable to produce useless pain of any used, in a large majority of cases. Most of the key instruments that I have seen are defective—the fulcrum being much too long. Most operators try to obviate the difficulty by lengthening the hook to agree with it; but it is a mistake: the fulcrum should be shortened, so that there should be as little spare room in the instrument when it is placed on a tooth, as possible. This will prevent the fulcrum from slipping down too low, in the effort to extract. The following will show the applicability of the key to a tooth. Place the fulcrum exactly on the line where the gum joins the tooth, then pass the hook over to the opposite side, and press the point down as low on the neck as possible. This will bring it below the fulcrum and give it the power to elevate. If the operator can then extract the tooth without allowing the fulcrum to slip down on the gum, the instrument is correct, and will, if skilfully used, always produce a happy effect. I have a large assortment of the various instruments now in use for extracting teeth, and in a large majority of cases I use my key, without any of the mangling effects generally produced by the instrument. It is a fact, that an instrument that cannot be used without bruising the gum and splitting the alveoli, ought not to be used at all; but I think a good turn-key is indispensable in a dentist's case; for there are some instances in which it is better, in my opinion, than any instrument I ever saw. It is to be hoped, therefore, that the key will be improved rather than abandoned.

